# *catena*-Poly[[[aqua­(2,2′-bipyridine-κ^2^ *N*,*N*′)zinc]-μ-furan-2,5-dicarboxyl­ato-κ^2^ *O* ^2^:*O* ^5^] dihydrate]

**DOI:** 10.1107/S1600536812045503

**Published:** 2012-11-10

**Authors:** Ya-Feng Li, Yue Xu, Xiao-Lin Qin, Yong-Peng Yuan, Wen-Yuan Gao

**Affiliations:** a School of Chemical Engineering, Changchun University of Technology, Changchun 130012, People’s Republic of China

## Abstract

In the title hydrated coordination polymer, {[Zn(C_6_H_2_O_5_)(C_10_H_8_N_2_)(H_2_O)]·2H_2_O}_*n*_, an infinite [1-10] chain is formed by the linking of [Zn(C_10_H_8_N_2_)(H_2_O)]^2+^ entities by bridging, monodentate furan-2,5-dicarboxyl­ate dianionic linkers. The Zn^2+^ coordination geometry is a trigonal bipyramid, with one N atom (from 2,2′-bipyridine) and one O atom (from the bridging dianion) in the axial positions. For each Zn^II^ atom, the dihedral angle between the furan ring of its coordinated bridging ligand and its coordinated bipyridine ring system is 87.19 (8)°. O—H⋯O hydrogen bonds involving both the coordinated and uncoordinated water mol­ecules generate a layer motif parallel to (001).

## Related literature

For a related structure, see: Li, *et al.* (2012 ▶).
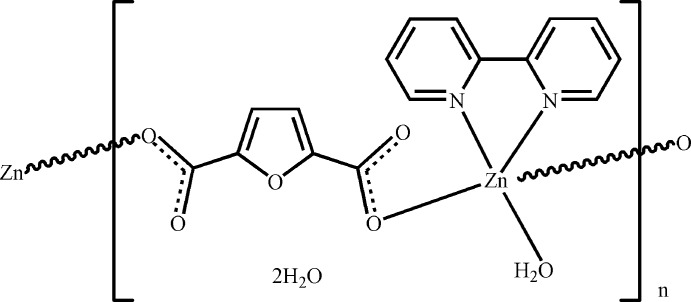


## Experimental

### 

#### Crystal data

[Zn(C_6_H_2_O_5_)(C_10_H_8_N_2_)(H_2_O)]·2H_2_O
*M* *_r_* = 429.70
Triclinic, 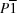

*a* = 8.5815 (17) Å
*b* = 9.2928 (19) Å
*c* = 12.753 (3) Å
α = 69.99 (3)°
β = 87.63 (3)°
γ = 65.85 (3)°
*V* = 866.2 (3) Å^3^
*Z* = 2
Mo *K*α radiation
μ = 1.47 mm^−1^
*T* = 293 K
0.43 × 0.34 × 0.23 mm

#### Data collection

Rigaku R-AXIS RAPID diffractometer
Absorption correction: multi-scan (*ABSCOR*; Higashi, 1995 ▶) *T*_min_ = 0.57, *T*_max_ = 0.73
8557 measured reflections
3925 independent reflections
3433 reflections with *I* > 2σ(*I*)
*R*_int_ = 0.023

#### Refinement

*R*[*F* ^2^ > 2σ(*F* ^2^)] = 0.036
*wR*(*F* ^2^) = 0.102
*S* = 1.10
3925 reflections
262 parameters
9 restraints
H atoms treated by a mixture of independent and constrained refinement
Δρ_max_ = 0.67 e Å^−3^
Δρ_min_ = −0.59 e Å^−3^

### 

Data collection: *PROCESS-AUTO* (Rigaku, 1998 ▶); cell refinement: *PROCESS-AUTO*; data reduction: Crystal Structure (Rigaku/MSC, 2002 ▶); program(s) used to solve structure: *SHELXS97* (Sheldrick, 2008 ▶); program(s) used to refine structure: *SHELXL97* (Sheldrick, 2008 ▶); molecular graphics: *DIAMOND* (Brandenburg, 2000 ▶); software used to prepare material for publication: *SHELXL97*.

## Supplementary Material

Crystal structure: contains datablock(s) I, global. DOI: 10.1107/S1600536812045503/hb6980sup1.cif

Structure factors: contains datablock(s) I. DOI: 10.1107/S1600536812045503/hb6980Isup2.hkl

Additional supplementary materials:  crystallographic information; 3D view; checkCIF report

## Figures and Tables

**Table 1:** Selected bond lengths (Å)

Zn1—O4^i^	2.0180 (17)
Zn1—O1	2.0221 (17)
Zn1—N2	2.078 (2)
Zn1—O1*W*	2.1142 (17)
Zn1—N1	2.1298 (19)

Symmetry code: (i) 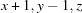.

**Table 2:** Hydrogen-bond geometry (Å, °)

*D*—H⋯*A*	*D*—H	H⋯*A*	*D*⋯*A*	*D*—H⋯*A*
O1*W*—H1*A*⋯O5^ii^	0.87 (2)	1.89 (2)	2.724 (2)	159 (2)
O1*W*—H1*B*⋯O1^iii^	0.86 (2)	1.88 (2)	2.701 (3)	158 (2)
O2*W*—H2*A*⋯O2	0.89 (2)	1.91 (3)	2.731 (4)	152 (5)
O2*W*—H2*B*⋯O5^iv^	0.89 (2)	2.12 (3)	2.902 (4)	146 (5)
O3*W*—H3*A*⋯O4	0.88 (2)	2.27 (3)	3.050 (4)	148 (4)
O3*W*—H3*B*⋯O2*W*	0.87 (2)	2.15 (3)	2.943 (7)	152 (5)

Symmetry codes: (ii) 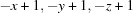; (iii) 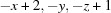; (iv) 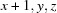.

**Fig. 1.**
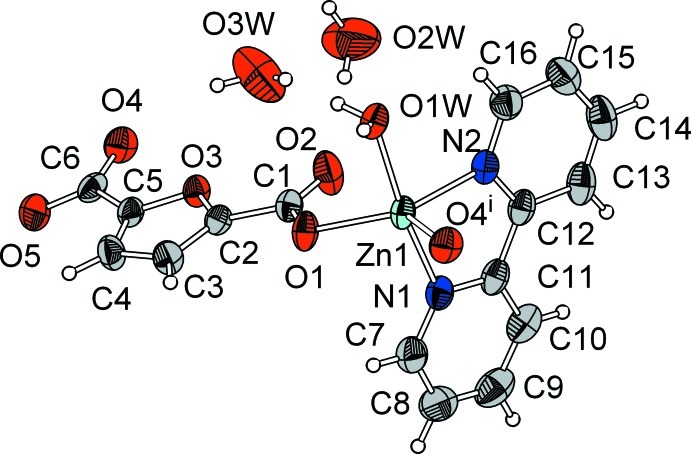
The unit cell of (I), showing displacement ellipsoids at the 50% probability level. [Symmetry code: (i) 1 + x, -1 + y, z.]

**Fig. 2.**
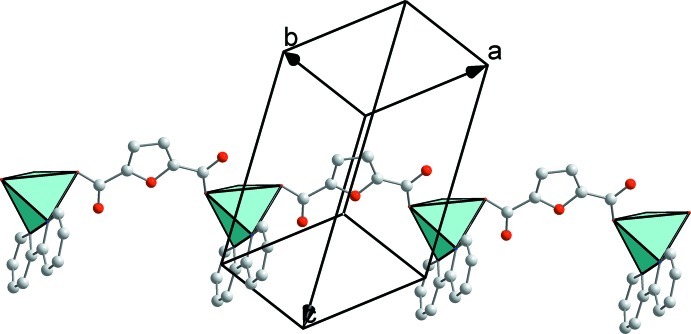
The polyhedral plot of (I), displaying the infinite chain formed by linking the adjacent Zn cations with furan-2,5-dicarboxylate.

**Fig. 3.**
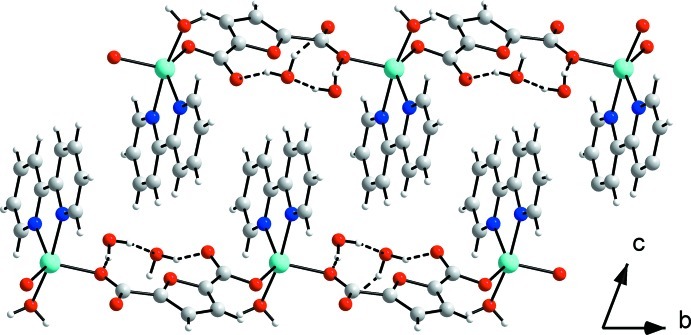
The ball-stick packing diagram of (I). The adjacent chains are holded together by the Owater–H···O H-bonding interactions to the supermolecular net.

## supplementary crystallographic information

### Comment

As the analogous structure of BDC (benzene-1,4-dicarboxyl acid), FDA (furan-2,5-dicarboxyl acid) attracts attentions owing to the bond angle of two carboxyl groups about 126° Recently, we utilized furan-2,5-dicarboxyl acid as the ligand to construct coordination polymers (Li, *et al.*, 2012). As an extension of this work, a chainlike compound, [Zn(H_2_O)(C_10_H_8_N_2_)(C_6_H_2_O_5_)]^.^2H_2_O (I), is now determined.

The asymmetric unit of (I) is consisted of one Zn(II) cation, one furan-2,5-dicarboxylate anion, one 2,2'-bipyridine and three waters involving in one coordinated waters and two structural water (Fig.1). Zn cation is coordinated by two N atoms of 2,2'-bipyridine, one water O atoms and two carboxylate O atoms, exhibiting a triangle bipyramid geometry (Table 1) with one O atoms of furan-2,5-dicarboxylate and one nitrogen atom of 2,2'-bipyridine in the axial positions. The adjacent Zn cations are connected by the furan-2,5-dicarboxylate to infinite chain (Fig.2). O_water_—H···O hydrogen bonds (Table 2) help to consolidate the structure.

### Experimental

In a typically synthesized route of (I), furan-2,5-dicarboxyl acid (0.0156 g, 0.10 mmol), Zn(NO_3_)_2_^.^6H_2_O (0.0300 g, 0.10 mmol), and 2,2'-bipyridine (0.0156, 0.10 mmol) and NaOH (0.004, 0.10 mmol) were dissolved in water (5 ml, 278 mmol) under stirring. The mixture with molar ratio of 1 (furan-2,5-dicarboxyl acid): 1 (Zn(NO_3_)_2_^.^6H_2_O): 1 (2,2'-bipyridine): 1 NaOH: 2780 H_2_O was layed under room temperature for 5 days. The colorless block product was collected as a single phase.

### Refinement

Water H atoms were located in a difference Fourier map and refined with O—H = 0.87 (2) Å and *U*_iso_(H) = 1.2Ueq(O). The carbon H-atoms were placed in calculated positions (C—H (furan and pyridine ring) = 0.93 Å) and were included in the refinement in the riding-model approximation, with *U*_iso_(H) = 1.2Ueq(C).

### Crystal data

**Table:**

[Zn(C_6_H_2_O_5_)(C_10_H_8_N_2_)(H_2_O)]·2H_2_O	*Z* = 2
*M**_r_* = 429.70	*F*(000) = 440
Triclinic, *P*1	*D*_x_ = 1.648 Mg m^−^^3^
Hall symbol: -P 1	Mo *K*α radiation, λ = 0.71073 Å
*a* = 8.5815 (17) Å	Cell parameters from 2000 reflections
*b* = 9.2928 (19) Å	θ = 3.3–27.5°
*c* = 12.753 (3) Å	µ = 1.47 mm^−^^1^
α = 69.99 (3)°	*T* = 293 K
β = 87.63 (3)°	Block, colorless
γ = 65.85 (3)°	0.43 × 0.34 × 0.23 mm
*V* = 866.2 (3) Å^3^	

### Data collection

**Table:**

Rigaku R-AXIS RAPID diffractometer	3925 independent reflections
Radiation source: fine-focus sealed tube	3433 reflections with *I* > 2σ(*I*)
Graphite monochromator	*R*_int_ = 0.023
Detector resolution: 10.00 pixels mm^-1^	θ_max_ = 27.5°, θ_min_ = 3.3°
ω scans	*h* = −10→11
Absorption correction: multi-scan (*ABSCOR*; Higashi, 1995)	*k* = −11→12
*T*_min_ = 0.57, *T*_max_ = 0.73	*l* = −16→16
8557 measured reflections	

### Refinement

**Table:**

Refinement on *F*^2^	Primary atom site location: structure-invariant direct methods
Least-squares matrix: full	Secondary atom site location: difference Fourier map
*R*[*F*^2^ > 2σ(*F*^2^)] = 0.036	Hydrogen site location: inferred from neighbouring sites
*wR*(*F*^2^) = 0.102	H atoms treated by a mixture of independent and constrained refinement
*S* = 1.10	*w* = 1/[σ^2^(*F*_o_^2^) + (0.0684*P*)^2^] where *P* = (*F*_o_^2^ + 2*F*_c_^2^)/3
3925 reflections	(Δ/σ)_max_ < 0.001
262 parameters	Δρ_max_ = 0.67 e Å^−^^3^
9 restraints	Δρ_min_ = −0.59 e Å^−^^3^

### Special details

**Table:**

Geometry. All e.s.d.'s (except the e.s.d. in the dihedral angle between two l.s. planes) are estimated using the full covariance matrix. The cell e.s.d.'s are taken into account individually in the estimation of e.s.d.'s in distances, angles and torsion angles; correlations between e.s.d.'s in cell parameters are only used when they are defined by crystal symmetry. An approximate (isotropic) treatment of cell e.s.d.'s is used for estimating e.s.d.'s involving l.s. planes.
Refinement. Refinement of *F*^2^ against ALL reflections. The weighted *R*-factor *wR* and goodness of fit *S* are based on *F*^2^, conventional *R*-factors *R* are based on *F*, with *F* set to zero for negative *F*^2^. The threshold expression of *F*^2^ > σ(*F*^2^) is used only for calculating *R*-factors(gt) *etc*. and is not relevant to the choice of reflections for refinement. *R*-factors based on *F*^2^ are statistically about twice as large as those based on *F*, and *R*- factors based on ALL data will be even larger.

### Fractional atomic coordinates and isotropic or equivalent isotropic displacement parameters (Å^2^)

**Table:**

	*x*	*y*	*z*	*U*_iso_*/*U*_eq_	
Zn1	1.02440 (3)	0.08858 (3)	0.301753 (18)	0.03278 (11)	
O1	0.8000 (2)	0.15494 (19)	0.36940 (13)	0.0406 (3)	
O2	0.7541 (3)	0.4185 (2)	0.26829 (18)	0.0583 (5)	
O3	0.44358 (18)	0.53732 (17)	0.35155 (12)	0.0331 (3)	
O4	0.1984 (2)	0.84783 (19)	0.33670 (14)	0.0437 (4)	
O5	0.0295 (2)	0.7310 (2)	0.43028 (18)	0.0558 (5)	
N1	0.9017 (2)	0.1152 (2)	0.15016 (15)	0.0381 (4)	
N2	1.1346 (2)	0.2204 (2)	0.18046 (15)	0.0366 (4)	
C1	0.7097 (3)	0.3148 (3)	0.33458 (17)	0.0351 (4)	
C2	0.5420 (3)	0.3677 (2)	0.38022 (17)	0.0308 (4)	
C3	0.4610 (3)	0.2801 (3)	0.4468 (2)	0.0401 (5)	
H3	0.5014	0.1632	0.4773	0.048*	
C4	0.3028 (3)	0.3994 (3)	0.4615 (2)	0.0427 (5)	
H4	0.2189	0.3765	0.5034	0.051*	
C5	0.2977 (3)	0.5523 (3)	0.40305 (17)	0.0329 (4)	
C6	0.1649 (3)	0.7242 (3)	0.38908 (18)	0.0355 (4)	
C7	0.7801 (3)	0.0635 (3)	0.1426 (2)	0.0494 (6)	
H7	0.7456	0.0075	0.2082	0.059*	
C8	0.7031 (4)	0.0910 (4)	0.0391 (3)	0.0573 (7)	
H8	0.6182	0.0545	0.0354	0.069*	
C9	0.7551 (4)	0.1728 (4)	−0.0565 (2)	0.0593 (7)	
H9	0.7053	0.1926	−0.1263	0.071*	
C10	0.8824 (4)	0.2266 (3)	−0.0497 (2)	0.0492 (6)	
H10	0.9190	0.2821	−0.1143	0.059*	
C11	0.9536 (3)	0.1952 (3)	0.05628 (18)	0.0387 (5)	
C12	1.0886 (3)	0.2478 (3)	0.07396 (18)	0.0373 (4)	
C13	1.1640 (3)	0.3239 (3)	−0.0142 (2)	0.0491 (6)	
H13	1.1321	0.3414	−0.0878	0.059*	
C14	1.2854 (4)	0.3726 (3)	0.0090 (2)	0.0558 (6)	
H14	1.3368	0.4232	−0.0489	0.067*	
C15	1.3307 (4)	0.3460 (3)	0.1186 (2)	0.0524 (6)	
H15	1.4113	0.3799	0.1359	0.063*	
C16	1.2541 (3)	0.2686 (3)	0.2017 (2)	0.0465 (5)	
H16	1.2862	0.2485	0.2759	0.056*	
O1W	1.1508 (2)	0.1017 (2)	0.43471 (13)	0.0397 (3)	
H1A	1.079 (3)	0.175 (3)	0.462 (2)	0.048*	
H1B	1.189 (3)	0.005 (2)	0.4890 (18)	0.048*	
O2W	0.8859 (7)	0.6378 (6)	0.2753 (2)	0.1299 (15)	
H2A	0.872 (8)	0.543 (5)	0.291 (4)	0.156*	
H2B	0.919 (8)	0.632 (7)	0.342 (3)	0.156*	
O3W	0.5178 (5)	0.8678 (5)	0.2312 (3)	0.1173 (12)	
H3A	0.456 (5)	0.842 (6)	0.285 (3)	0.141*	
H3B	0.612 (4)	0.777 (5)	0.240 (4)	0.141*	

### Atomic displacement parameters (Å^2^)

**Table:**

	*U*^11^	*U*^22^	*U*^33^	*U*^12^	*U*^13^	*U*^23^
Zn1	0.03146 (16)	0.02874 (14)	0.03086 (15)	−0.00672 (10)	0.00895 (10)	−0.01011 (10)
O1	0.0337 (8)	0.0338 (7)	0.0378 (8)	−0.0014 (6)	0.0106 (6)	−0.0102 (7)
O2	0.0560 (11)	0.0468 (9)	0.0676 (12)	−0.0222 (9)	0.0314 (10)	−0.0170 (9)
O3	0.0296 (7)	0.0248 (6)	0.0381 (7)	−0.0058 (6)	0.0078 (6)	−0.0109 (6)
O4	0.0395 (9)	0.0297 (7)	0.0515 (9)	−0.0041 (6)	0.0042 (7)	−0.0152 (7)
O5	0.0394 (10)	0.0562 (10)	0.0831 (13)	−0.0156 (8)	0.0249 (9)	−0.0454 (11)
N1	0.0325 (9)	0.0392 (9)	0.0380 (9)	−0.0089 (8)	0.0072 (7)	−0.0165 (8)
N2	0.0384 (10)	0.0324 (8)	0.0339 (9)	−0.0114 (7)	0.0099 (7)	−0.0108 (8)
C1	0.0311 (10)	0.0355 (10)	0.0346 (10)	−0.0092 (9)	0.0064 (8)	−0.0140 (9)
C2	0.0286 (10)	0.0249 (8)	0.0335 (9)	−0.0053 (7)	0.0036 (8)	−0.0116 (8)
C3	0.0385 (12)	0.0266 (9)	0.0505 (12)	−0.0106 (9)	0.0113 (10)	−0.0128 (9)
C4	0.0360 (12)	0.0384 (11)	0.0554 (13)	−0.0154 (9)	0.0186 (10)	−0.0205 (11)
C5	0.0284 (10)	0.0323 (9)	0.0373 (10)	−0.0083 (8)	0.0072 (8)	−0.0175 (9)
C6	0.0317 (11)	0.0335 (10)	0.0404 (11)	−0.0052 (8)	0.0028 (8)	−0.0225 (9)
C7	0.0407 (13)	0.0544 (14)	0.0549 (14)	−0.0168 (11)	0.0118 (11)	−0.0263 (13)
C8	0.0395 (14)	0.0617 (16)	0.0741 (18)	−0.0131 (12)	0.0014 (12)	−0.0378 (16)
C9	0.0544 (17)	0.0597 (16)	0.0537 (15)	−0.0058 (13)	−0.0076 (13)	−0.0294 (14)
C10	0.0542 (15)	0.0433 (12)	0.0390 (12)	−0.0084 (11)	0.0010 (10)	−0.0162 (11)
C11	0.0384 (12)	0.0325 (10)	0.0347 (10)	−0.0039 (9)	0.0059 (9)	−0.0133 (9)
C12	0.0389 (12)	0.0288 (9)	0.0334 (10)	−0.0051 (8)	0.0083 (8)	−0.0102 (8)
C13	0.0528 (15)	0.0445 (12)	0.0349 (11)	−0.0125 (11)	0.0143 (10)	−0.0073 (10)
C14	0.0545 (16)	0.0459 (13)	0.0536 (15)	−0.0193 (12)	0.0230 (12)	−0.0062 (12)
C15	0.0469 (15)	0.0481 (13)	0.0614 (16)	−0.0234 (12)	0.0133 (12)	−0.0152 (13)
C16	0.0487 (14)	0.0474 (12)	0.0450 (12)	−0.0217 (11)	0.0098 (10)	−0.0168 (11)
O1W	0.0422 (9)	0.0358 (7)	0.0343 (8)	−0.0091 (7)	0.0081 (6)	−0.0140 (7)
O2W	0.221 (4)	0.178 (4)	0.0612 (16)	−0.155 (4)	0.025 (2)	−0.038 (2)
O3W	0.091 (2)	0.135 (3)	0.123 (3)	−0.069 (2)	0.019 (2)	−0.017 (2)

### Geometric parameters (Å, º)

**Table:**

Zn1—O4^i^	2.0180 (17)	C7—H7	0.9300
Zn1—O1	2.0221 (17)	C8—C9	1.364 (5)
Zn1—N2	2.078 (2)	C8—H8	0.9300
Zn1—O1W	2.1142 (17)	C9—C10	1.391 (4)
Zn1—N1	2.1298 (19)	C9—H9	0.9300
O1—C1	1.281 (3)	C10—C11	1.392 (3)
O2—C1	1.226 (3)	C10—H10	0.9300
O3—C5	1.367 (2)	C11—C12	1.481 (3)
O3—C2	1.369 (2)	C12—C13	1.397 (3)
O4—C6	1.255 (3)	C13—C14	1.371 (4)
O5—C6	1.242 (3)	C13—H13	0.9300
N1—C7	1.334 (3)	C14—C15	1.378 (4)
N1—C11	1.342 (3)	C14—H14	0.9300
N2—C12	1.338 (3)	C15—C16	1.371 (4)
N2—C16	1.344 (3)	C15—H15	0.9300
C1—C2	1.483 (3)	C16—H16	0.9300
C2—C3	1.345 (3)	O1W—H1A	0.873 (16)
C3—C4	1.413 (3)	O1W—H1B	0.864 (16)
C3—H3	0.9300	O2W—H2A	0.890 (19)
C4—C5	1.343 (3)	O2W—H2B	0.890 (19)
C4—H4	0.9300	O3W—H3A	0.879 (19)
C5—C6	1.488 (3)	O3W—H3B	0.871 (19)
C7—C8	1.395 (4)		
			
O4^i^—Zn1—O1	123.96 (7)	O5—C6—C5	116.0 (2)
O4^i^—Zn1—N2	101.59 (8)	O4—C6—C5	117.87 (19)
O1—Zn1—N2	134.31 (7)	O4—C6—C5	117.87 (19)
O4^i^—Zn1—O1W	89.61 (7)	N1—C7—C8	121.8 (3)
O1—Zn1—O1W	91.03 (7)	N1—C7—H7	119.1
N2—Zn1—O1W	92.77 (7)	C8—C7—H7	119.1
O4^i^—Zn1—N1	96.59 (8)	C9—C8—C7	118.5 (3)
O1—Zn1—N1	92.21 (7)	C9—C8—H8	120.7
N2—Zn1—N1	78.06 (8)	C7—C8—H8	120.7
O1W—Zn1—N1	169.79 (7)	C8—C9—C10	120.1 (2)
C1—O1—Zn1	112.97 (14)	C8—C9—H9	119.9
C5—O3—C2	105.96 (16)	C10—C9—H9	119.9
C6—O4—Zn1^ii^	122.19 (15)	C9—C10—C11	118.3 (3)
C7—N1—C11	119.8 (2)	C9—C10—H10	120.8
C7—N1—Zn1	125.87 (17)	C11—C10—H10	120.8
C11—N1—Zn1	114.32 (15)	N1—C11—C10	121.3 (2)
C12—N2—C16	119.0 (2)	N1—C11—C12	115.47 (19)
C12—N2—Zn1	115.74 (15)	C10—C11—C12	123.2 (2)
C16—N2—Zn1	125.13 (16)	N2—C12—C13	120.9 (2)
O2—C1—O1	124.2 (2)	N2—C12—C11	116.2 (2)
O2—C1—O1	124.2 (2)	C13—C12—C11	122.9 (2)
O2—C1—C2	121.42 (19)	C14—C13—C12	119.4 (2)
O2—C1—C2	121.42 (19)	C14—C13—H13	120.3
O1—C1—C2	114.39 (19)	C12—C13—H13	120.3
C3—C2—O3	110.11 (18)	C13—C14—C15	119.4 (2)
C3—C2—C1	132.51 (18)	C13—C14—H14	120.3
O3—C2—C1	117.38 (18)	C15—C14—H14	120.3
C2—C3—C4	106.94 (19)	C16—C15—C14	118.5 (2)
C2—C3—H3	126.5	C16—C15—H15	120.7
C4—C3—H3	126.5	C14—C15—H15	120.7
C5—C4—C3	106.4 (2)	N2—C16—C15	122.8 (2)
C5—C4—H4	126.8	N2—C16—H16	118.6
C3—C4—H4	126.8	C15—C16—H16	118.6
C4—C5—O3	110.58 (18)	Zn1—O1W—H1A	110.7 (18)
C4—C5—C6	130.7 (2)	Zn1—O1W—H1B	109.3 (17)
O3—C5—C6	118.72 (18)	H1A—O1W—H1B	106 (2)
O5—C6—O4	126.2 (2)	H2A—O2W—H2B	103 (3)
O5—C6—O4	126.2 (2)	H3A—O3W—H3B	108 (3)
			
O4^i^—Zn1—O1—C1	−179.73 (13)	C2—O3—C5—C4	−0.2 (2)
N2—Zn1—O1—C1	−4.84 (19)	C2—O3—C5—C6	178.97 (17)
O1W—Zn1—O1—C1	90.05 (15)	O4—O4—C6—O5	0.00 (18)
N1—Zn1—O1—C1	−80.27 (15)	Zn1^ii^—O4—C6—O5	−3.8 (3)
O4^i^—Zn1—N1—C7	82.2 (2)	O4—O4—C6—C5	0.00 (19)
O1—Zn1—N1—C7	−42.31 (19)	Zn1^ii^—O4—C6—C5	175.53 (13)
N2—Zn1—N1—C7	−177.3 (2)	C4—C5—C6—O5	−6.4 (3)
O1W—Zn1—N1—C7	−150.7 (3)	O3—C5—C6—O5	174.61 (18)
O4^i^—Zn1—N1—C11	−99.23 (15)	C4—C5—C6—O4	174.2 (2)
O1—Zn1—N1—C11	136.22 (15)	O3—C5—C6—O4	−4.8 (3)
N2—Zn1—N1—C11	1.27 (14)	C4—C5—C6—O4	174.2 (2)
O1W—Zn1—N1—C11	27.8 (4)	O3—C5—C6—O4	−4.8 (3)
O4^i^—Zn1—N2—C12	90.59 (16)	C11—N1—C7—C8	−0.4 (4)
O1—Zn1—N2—C12	−85.08 (17)	Zn1—N1—C7—C8	178.01 (18)
O1W—Zn1—N2—C12	−179.24 (15)	N1—C7—C8—C9	0.2 (4)
N1—Zn1—N2—C12	−3.79 (14)	C7—C8—C9—C10	0.1 (4)
O4^i^—Zn1—N2—C16	−84.86 (19)	C8—C9—C10—C11	−0.2 (4)
O1—Zn1—N2—C16	99.5 (2)	C7—N1—C11—C10	0.3 (3)
O1W—Zn1—N2—C16	5.32 (19)	Zn1—N1—C11—C10	−178.29 (17)
N1—Zn1—N2—C16	−179.24 (19)	C7—N1—C11—C12	179.80 (19)
O2—O2—C1—O1	0.00 (12)	Zn1—N1—C11—C12	1.2 (2)
O2—O2—C1—C2	0.00 (9)	C9—C10—C11—N1	0.0 (3)
Zn1—O1—C1—O2	−0.9 (3)	C9—C10—C11—C12	−179.4 (2)
Zn1—O1—C1—O2	−0.9 (3)	C16—N2—C12—C13	0.5 (3)
Zn1—O1—C1—C2	178.44 (13)	Zn1—N2—C12—C13	−175.20 (16)
C5—O3—C2—C3	0.1 (2)	C16—N2—C12—C11	−178.66 (19)
C5—O3—C2—C1	179.71 (16)	Zn1—N2—C12—C11	5.6 (2)
O2—C1—C2—C3	175.1 (2)	N1—C11—C12—N2	−4.5 (3)
O2—C1—C2—C3	175.1 (2)	C10—C11—C12—N2	175.0 (2)
O1—C1—C2—C3	−4.2 (3)	N1—C11—C12—C13	176.33 (19)
O2—C1—C2—O3	−4.4 (3)	C10—C11—C12—C13	−4.2 (3)
O2—C1—C2—O3	−4.4 (3)	N2—C12—C13—C14	−0.7 (3)
O1—C1—C2—O3	176.30 (17)	C11—C12—C13—C14	178.5 (2)
O3—C2—C3—C4	0.0 (3)	C12—C13—C14—C15	−0.1 (4)
C1—C2—C3—C4	−179.5 (2)	C13—C14—C15—C16	1.1 (4)
C2—C3—C4—C5	−0.1 (3)	C12—N2—C16—C15	0.5 (4)
C3—C4—C5—O3	0.2 (3)	Zn1—N2—C16—C15	175.76 (19)
C3—C4—C5—C6	−178.8 (2)	C14—C15—C16—N2	−1.3 (4)

Symmetry codes: (i) *x*+1, *y*−1, *z*; (ii) *x*−1, *y*+1, *z*.

### Hydrogen-bond geometry (Å, º)

**Table:**

*D*—H···*A*	*D*—H	H···*A*	*D*···*A*	*D*—H···*A*
O1*W*—H1*A*···O5^iii^	0.87 (2)	1.89 (2)	2.724 (2)	159 (2)
O1*W*—H1*B*···O1^iv^	0.86 (2)	1.88 (2)	2.701 (3)	158 (2)
O2*W*—H2*A*···O2	0.89 (2)	1.91 (3)	2.731 (4)	152 (5)
O2*W*—H2*B*···O5^v^	0.89 (2)	2.12 (3)	2.902 (4)	146 (5)
O3*W*—H3*A*···O4	0.88 (2)	2.27 (3)	3.050 (4)	148 (4)
O3*W*—H3*B*···O2*W*	0.87 (2)	2.15 (3)	2.943 (7)	152 (5)

Symmetry codes: (iii) −*x*+1, −*y*+1, −*z*+1; (iv) −*x*+2, −*y*, −*z*+1; (v) *x*+1, *y*, *z*.
